# Dr. Slayton’s Gutta Percha

**Published:** 1856-12

**Authors:** 


### DR. SLAYTON'S GUTTA PERCHA.

It seemed just the thing for temporary sets, and full of promise for future improvement—beautiful gum color, which stood well for months in the mouth of the inventor—can learn to use it in an hour, and make a full set on short notice, and besides, it feels so comfortable in the mouth, and can be so easily altered as the alveolar arch changes, and draws so much more gently than gold on the operator's pocket, even at $40 per pound. Taking, decidedly.

Patent applied for, etc. Ah, why throw this restriction around it—why not manufacture and sell to all at a reasonable price—$40 for what costs $10—$50 for the glorious privilege of using this grand improvement! Ahem, but we intend to keep it in good society, and out of the hands of tinkers and peddlers, as they would ruin it directly. We have an arrangement with an Eastern house, of world-wide reputation for its great moral worth, so it will be manufactured all right, and probably be improved, and they will in no case sell to any but those who have purchased the right—so you see it is all safe. We are denizens of the mighty valley of the Mississippi, where nature has done things on a grand scale, and the people are progressive Americans. If this thing is good, all right—if not, $50 or $60 would not harm us much, and besides, we love to be humbugged, just for variety occasionally; so hurrah for the "right."

Subsequently, to be armed to the teeth for all emergencies, a fresh supply is obtained—the package opened, hoping to see signs of improvement. Whew! what's up now?—not the genuine article; differs in toto from the original—$50 for the pre-emption right to buy this stuff! Well, we couldn't help it—the great N. Y. Teeth Manufacturing Co., 404 Broadway, are wickedly and with malice prepense, selling a preparation similar to it for $20, and it don't pay to make it like the first at that rate. Cool, isn't it?

Tempus fugit. Gentlemen outsiders without pre-emption rights, no matter about your paying your $30 or $50, but please walk up and *try* a little gutta percha.

The inventor, his partners in selling rights to buy and use, as well as the purchasers, are, no doubt, all good men and true: all having had the wool pulled over their eyes—the difference between them in the result being that while one class has plasters, the others have the *golden fleece.* Poor Gutta Percha! Requiescat. SOUTH.

